# Supporting the Process of Help-Seeking by Caregivers of Functionally Dependent Older Persons Through Electronic Health: Protocol for a Multicenter Co-Design

**DOI:** 10.2196/11634

**Published:** 2019-04-26

**Authors:** Karine Latulippe, Manon Guay, Sophie Éthier, Andrée Sévigny, Véronique Dubé, Véronique Provencher, Valérie Poulin, Anick MC Giguere, Mélanie Tremblay, Maude Carignan, Dominique Giroux

**Affiliations:** 1 Department of Teaching and Learning Studies Laval University Quebec, QC Canada; 2 School of Rehabilitation University of Sherbrooke Sherbrooke, QC Canada; 3 Research Centre on Aging Centre Intégré Universitaire de Santé et de Services Sociaux de l’Estrie-Centre Hospitalier Universitaire de Sherbrooke Sherbrooke, QC Canada; 4 School of Social Work and Criminology Laval University Quebec, QC Canada; 5 Center of Excellence on Aging Quebec Quebec, QC Canada; 6 Faculty of Nursing University of Montreal Montreal, QC Canada; 7 Research Center University Hospital Center of Montreal Montreal, QC Canada; 8 Université du Québec in Trois-Rivières Trois-Rivières, QC Canada; 9 Interdisciplinary Center for Research in Rehabilitation and Social Integration Quebec, QC Canada; 10 Department of Family Medicine and Emergency Medicine Laval University Quebec, QC Canada; 11 Department of Rehabilitation Laval University Quebec, QC Canada

**Keywords:** caregivers, aged, help-seeking behavior, community-based participatory research, eHealth, telemedicine

## Abstract

**Background:**

It is often only when the initial signs of exhaustion appear that caregivers first may engage in help-seeking behavior, but it is difficult for them to know which is the most appropriate formal service in their situation. Electronic health (eHealth) can support caregivers in keeping the older person they are caring for at home, but few eHealth tools designed for supporting the process of help-seeking by caregivers of functionally impaired older persons have been developed using a co-design approach.

**Objective:**

This paper aims to describe the protocol of a project that tries to assist caregivers to target their needs and those of the older person they support early in their help-seeking process, and guide them effectively to the formal service most appropriate for their situation. This project aims to answer the following questions: (1) What type of tool can better support caregivers to identify their needs and those of the older person they are caring for and then refer them to an appropriate formal service? and (2) What information should be found in such a tool?

**Methods:**

This study presents a description of the process of an ongoing multicenter research project based on a co-design approach, which includes 3 phases (1) identification of caregivers’ needs in terms of tools to support their help-seeking behavior, (2) development of a tool, and (3) evaluation of its usability.

**Results:**

The project began in January 2016 with the ethics application for the 3 phases of the project. For phase 1, recruitment began in December 2016 and ended in September 2017. Phase 2 began in the spring of 2017 and ended in June 2018. All the co-design sessions have been completed. Phase 3 of the project will begin in September 2018.

**Conclusions:**

Although there are some challenges associated with this type of methodology, the methodology still remains relevant, as it involves future users in the development of a tool, which increases the chances that the tool will meet the users' needs.

**International Registered Report Identifier (IRRID):**

DERR1-10.2196/11634

## Introduction

### Background

Aging of the population leads to a reorganization of health and social services (HSS) for older persons because of the greater pressure on the HSS network. Keeping older persons in their homes as long as possible is economically and socially desirable [1]. Moreover, this wish is shared by the older persons themselves [2]. Nonetheless, this depends, in part, on caregivers [3]. A caregiver, in this study, refers to anyone who provides care and services to a functionally impaired older person on a voluntary and weekly basis [3]. Although it is gratifying for a number of caregivers, contributing to looking after older persons at home is a task that can prove demanding on a day-to-day basis. Caregivers feel poorly equipped to assume this role, which is described by some as a moral responsibility [4]. Moreover, success in home care for older persons depends on the capacity to respond to the needs of those suffering a loss of independence [5,6]. There are many risks for the older person when the support is inadequate or when the burden is too great [7]. Reinhard et al [7] report such potential risks as (1) abuse, (2) medication errors, (3) negligence, and (4) conflicts with the caregiver.

Electronic health (eHealth) can support caregivers in keeping the older person they are caring for at home [8]. Indeed, a number of arguments support the idea of turning to eHealth to help caregivers of functionally impaired older persons in their role. These include acceptability [9], the current use of the internet [10], fewer problems related to moves and respite [8], reduced costs [11], its availability at all times and in all locations [12], the possibilities of using a variety of pedagogical modalities [13], and the efficacy of this type of intervention [14].

Telemedicine, tele-assistance, assistive technologies, communication-linked technologies, tracking systems, Web-based services, and mobile apps [15] are among the various types of eHealth tools for caregivers. Few of these tools were developed with a co-design approach. In addition, very few eHealth tools developed for caregivers have specifically focused on the process of seeking help. It is often only when the initial signs of exhaustion appear that caregivers first undertake the process of help-seeking behavior, but it is difficult for them to know which is the most appropriate formal service in their situation, without assistance from HSS professionals [7].

With the objective of developing an eHealth tool, which supports the process of caregivers’ help-seeking behavior, a review of the literature was conducted to identify different theoretical models that could support this process.

### Theoretical Frame

We found a number of works bearing on modeling of the process of help-seeking [16-25]. Nevertheless, there is no consensus on the greater relevance of any 1 model [25]. *Levkoff’s help-seeking behavior model for dementia* seems to correspond to the process of help-seeking behavior for the caregiver until the latter contacts a care provider [22]. Indeed, this model is specific to the help-seeking behavior for dementia and includes 4 components: the illness and the experience of symptoms, evaluation of symptoms, the decision to look for formal services, and contact with care providers. Recognizing the symptoms (either by the one being helped or by the caregiver) begins the process of help-seeking behavior. Subsequently, the caregiver must interpret the symptoms (using cognitive and sensory capacities), evaluating the degree of severity and the potential duration. The decision to seek help and to contact a care provider depends on a number of factors. To better understand the limitations of these last 2 steps, we identified in a previous study [26] 5 categories of factors influencing the search for assistance: informational factors, factors linked to the service, experiential factors, personal factors, and relational factors. Each stage of *Levkoff’s help-seeking behavior model for dementia* and the comprehension of factors limiting the recourse to formal services offer a potential transition where interventions could facilitate the process of assistance.

### Objectives

Consistent with this perspective, the purpose of this paper is to describe the protocol of a project that aims to assist caregivers to target early in their help-seeking process their needs and those of the older person they support and guide them effectively to the formal service most appropriate to their situation. The 3 objectives of the project are (1) identifying the needs of the caregivers in terms of tools to accompany their process of help-seeking behavior, (2) developing a tool for caregivers that corresponds to the needs they have expressed, and (3) evaluating the usability of the tool.

## Methods

### Research Design

**Figure 1:**
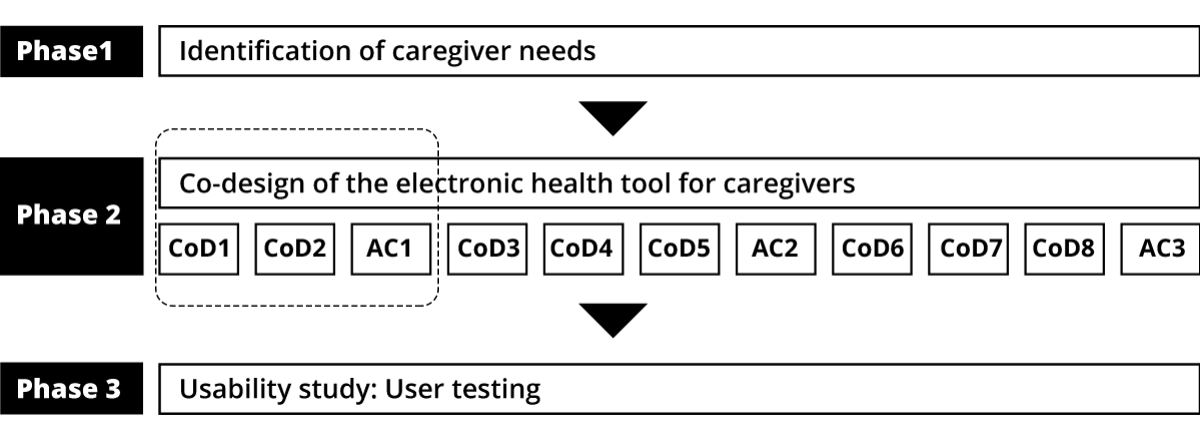
Phases of project. AC: advisory committee session; CoD: co-design session.

To attain these objectives, this study is based on a participatory design, more specifically, a co-design approach. Co-design is defined as the creation of useful knowledge and actions, in this case, an eHealth tool, which involves groups experiencing the issue, even in the research process; they assume simultaneously the role of creators, decision makers, and users [27]. Thus, caregivers, acting as designers, can intervene directly in their future eHealth tool and draw upon their knowledge to develop technologies that respect their needs and their ways of doing things [28]. This project has 3 phases (Figure 1). The objective of phase 1 is to identify the needs of caregivers of functionally impaired older persons. On the basis of the results from this phase, the objective of phase 2 will be to co-design an eHealth tool to support the help-seeking process of caregivers. Finally, phase 3 will be a usability study aimed to verify the results obtained with the co-design process.

### The Research Sites

The study takes place in 11 regions of Québec (Côte-Nord, Mauricie, Centre-du-Québec, Capitale-Nationale, Chaudière-Appalaches, Montérégie, Bas St-Laurent, Gaspésie, Outaouais, Montréal, and Laval). The meeting places vary, depending on the availability of locations (eg, municipal or community premises or those connected to the HSS network).

### Participants and Selection Criteria

The number of participants and the selection criteria for each group of participants are as follows.

1. Caregivers: The objective is to recruit a total of 50 caregivers. In the context of this project, any person who provides unremunerated assistance on a sustained (weekly) basis to a functionally impaired older person will be considered a caregiver.
2. Community workers: The goal is to have 30 members of community associations involved in this project. These must offer services or interact directly with caregivers of functionally impaired older persons.
3. Health and social service professionals (HSSP): The objective is to involve 30 professionals from the public sector of HSS. Like the community workers, these must offer services or interact directly with caregivers of functionally impaired older persons. They may be nurses, nursing assistants, client care attendants, home care workers, occupational therapists, physiotherapists, doctors, social workers, psychologists, or others.

### Recruitment

To have access to a diversity of perspectives, it is hoped that the participants will present a variety of characteristics, in terms of their profession (social worker, occupational therapist, physiotherapist, doctor, nurse, etc), their organization (administrative agency, association, organization, and other), and their sociodemographic attributes. A purposive sampling strategy will be used via advertising in local community centers, family medicine groups (FMGs), and community organizations. For the HSSP, direct contact will be made with the management of older persons services. The latter will target potential participants as a function of selection criteria, and the HSSP will establish contact with the research team. A network sampling approach will also be used as recruitment through advertising alone will be insufficient to reach all the types of participants targeted. Thus, community organizations (through a direct approach) and HSSP willing to recruit caregivers to participate in the study will be solicited. When caregivers express interest in participating in the study, HSSP and community workers will be asked to transmit their coordinates so that research agents could establish contact with them.

### The Research Team

All stages (recruitment, data collection, and analysis) will be done by 1 or more members of the research team. The research team is made up of the study director; a researcher in gerontology (DG); 2 doctoral students (KL and MT), one of who has experience in user experience and the other in participatory studies; and finally, a research professional with expertise in qualitative research (MC).

#### Phase 1 (Objective 1): Identify the Needs of Caregivers in Terms of Tools to Support Their Process of Help-Seeking Behavior

##### Online Questionnaire

###### Data Collection

A total of 2 distinct forms of data collection will be used to document caregivers’ needs for support in their process of seeking help. The first is targeted at community workers and those from the Québec HSS network. They will be consulted via an online questionnaire inspired by Levkoff’s help-seeking behavior model for dementia [22] (see Multimedia Appendix 1).

###### Analysis

Considering that the online questionnaire is essentially composed of open-ended questions, we will use the method of analytical questioning [29]. The NVivo software (QSR International) [30] will be used to facilitate this analysis.

###### Participants

The participation of 55 HSSP or community workers (approximately 5 participants per targeted region) or a number sufficient to reach the data saturation level is envisaged. The goal is, above all, to reach data saturation.

##### Individual Interviews

###### Data Collection

The second form of data collection consists of semidirected individual interviews with caregivers. The interview plan covers sociodemographic data (including a profile of the use of digital tools such as the internet, electronic tablets, and mobile phone); open-ended questions also based on Levkoff’s help-seeking behavior model for dementia [22]; and finally, a questionnaire related to the level of literacy, the All Aspects of Health Literacy Scale (AAHLS) [31] (see Multimedia Appendix 2). This measure is important to consider in the development of an eHealth instrument designed for a group, which is homogeneous in its role (in other words, all people who care for an older person) but heterogeneous in its characteristics (with widely varying ages and levels of education). The AAHLS has been validated in English for individuals aged between 18 and 65 years in face-to-face encounters but may be adapted to telephone interviews as it is a self-evaluation measure rather than a direct evaluation of capacities. With this measure, it is not a case of categorizing the levels of literacy but rather of developing a descriptive analysis of participants’ capacities [31]. The instrument was translated into French (a free translation) and tested beforehand.

###### Analysis

As for the online questionnaire, we will rely on analysis through analytical questioning [29]. The NVivo software [30] will be used to facilitate this analysis.

###### Participants

We expect the participation of 27 caregivers (approximately 2 to 3 participants per targeted region) or a sufficient number to reach the data saturation point.

#### Phase 2 (Objective 2): Develop a Tool for Caregivers That Meets the Needs They Have Expressed

##### Co-Design Workshops

In general, co-design includes the creation of a group comprising 8 to 12 people, who jointly develop an eHealth instrument over 3 to 5 co-design meetings, lasting between 90 min and a day [32-37]. Nonetheless, in the context of this project, it is, on the one hand, unrealistic to develop a tool supporting the process of help-seeking behavior in merely 5 working sessions. On the other hand, it is also unrealistic to involve caregivers in all the sessions of such a process, given their onerous responsibilities and lack of available time. In addition, it is important to include the perspective of a number of regions of Québec to take account of the differences in available resources. We, therefore, opted for a different methodology, that is, each co-design session will be composed of a different group. It consists of 8 co-design sessions (with participants of the 11 administrative regions targeted by the project), which will take place over a period of 13 months. The co-design sessions will last 3 hours and will be facilitated or moderated by the research team. Each co-design session is scheduled to continue the work of the previous session until a prototype is made. We aim to have approximately 1 month between sessions to allow the research team to analyze the data and prepare for the next session based on the results of the previous session and at the same time, respect the end of funding. These working sessions will be interspersed with meetings with an advisory committee (which also includes caregivers, community workers, and HSSP) whose mandate is to guide the progression of the prototype and ensure continuity, so that the material stemming from the working sessions is truly integrated into the prototype (Figure 1).

Co-design involves the use of tools and techniques, which combine narratives, creativity, and imagination [38]. A variety of methods will be used (group discussions, world café, individual work, collective sessions, and mock-ups) to ensure we reach all participants based on their individual characteristics and to guarantee that power is shared within the group. This process is intended to be iterative, varying from one session to the next, to cover all the issues effectively. The collaboration of a user experience expert will be necessary to direct the planning of objectives for each session. The results of phase 1 of the project will be presented to the co-designers to fully integrate knowledge about caregivers’ needs for support in their process of seeking help.

##### Data Collection

The data will be obtained from (1) notes taken by the team, (2) artifacts produced by each group, and (3) notes taken after the working sessions via a meeting with the research team to share their impressions. The co-design sessions and those of the advisory committee will be filmed to further develop certain aspects of other methods of data collection if necessary.

##### Analysis

An analysis by analytical questioning [29] will also be performed using NVivo software [30] to meet the objectives of each session. The final form of the tool (website, mobile app, etc) is unknown as it is the co-designers who will decide on its ultimate version.

##### Participants

We aim to recruit 6 to 10 participants in each co-design (caregivers, community workers, and HSSP) with a majority of caregivers to ensure a strong voice from this subgroup.

#### Phase 3 (Objective 3): Evaluating the Tool’s Usability and User Satisfaction

The third phase is planned to gauge the instrument’s usability. This is a matter of observing potential future users accomplish tasks using the tool, with the aim of identifying any potential problems. Furthermore, 2 distinct methods will be used for the study of usability [39] in the context of an individual encounter with the participant in a location of their choice, for a maximum of 1 hour. The 2 methods employed are the think-aloud approach, to measure usability, and the questionnaire, to measure user satisfaction.

##### Think-Aloud Method

First, in a process of digital tool development, the think-aloud method is frequently used to reveal usability problems that the user might encounter with the tool [39]. In general, this method aims to capture a systematic process of thinking aloud and analyze this process to gain a deeper appreciation of any problems, which could arise during the use of the digital tool [40]. As this think-aloud method can prove difficult for participants, a trial run will take place with a task similar to that which will be actually evaluated [40]. These will both take place after the tool is developed (phase 2).

##### Data Collection

The sessions will be filmed in a context, which includes the individual and the tool, as well as another screen, which allows the user to see directly what is happening with the tool. This will allow for the transcription of all the verbal data and permit us to associate them with the digital tool.

##### Analysis

The transcriptions will be coded to identify step by step how the person performs the task, as well as the problems encountered. The codes will be generated through an inductive approach [40].

##### Participants

The selection of a representative sample of potential users is crucial with this type of method, which includes those with a variety of skills. Although it is generally admitted that calling upon 5 participants is sufficient for a usability study, seeking the participation of 10 individuals with diverse perspectives increases the validity of the results by 25% [41]. Consequently, a purposive sample with 5 caregivers and 5 community workers or HSSP is our objective.

##### Questionnaire

###### Data Collection

As for user satisfaction (the second method), the most common method used is the questionnaire [39]. The standardized questionnaires used most often are those for user interaction satisfaction, the modified technology acceptance model questionnaire, and the International Business Machines Corporation (IBM) usability questionnaire [39]. The modified technology acceptance model questionnaire was conceived in a telemonitoring context and is less relevant to this study [42]. The questionnaire for user interaction satisfaction in its shorter version includes 20 questions with responses on a scale from 1 to 10. The purchase of a license is required. As for the IBM usability questionnaire [43], it contains 19 questions with responses on a scale from 1 to 7. This questionnaire is designed to be administered after the performance of the task, that is, immediately after the think-aloud method. It is the latter questionnaire, which was selected for this study.

###### Analysis

A descriptive analysis will be performed (means, percentage).

###### Participants

The participants will be the same who have done the think-aloud exercise. The questionnaire will be administered during the same session.

### Ethical Considerations

This project was approved by the Comité d'éthique de la recherche des Centres de santé et de services sociaux de la Vieille-Capitale (the Research Ethics Committee of the Health and Social Service Centres of the Old Capital). As this is a multicenter project, it also needed and received the approval of each research ethics committee of HSS network centers for the regions targeted through a formal agreement. There is no compensation offered for phase 1 of the project, as this stage does not involve any traveling. A monetary compensation of Can $20 for each participant is, however, planned for phases 2 and 3. There are no physical or moral risks to the study participants. However, it is possible that this could be inconvenient due to a required reorganization of the usual routine or supervision. Throughout the research, the raw data will be rendered anonymous before being analyzed. Only 2 research professionals will have access to the list containing the names and codes, which will be stored separately from the research material, data, and information and consent forms. All the research material, including the information and consent forms and the recordings, will be kept in a locked filing cabinet in a locked room. The digital data will be stored in encrypted files, access to which will be protected by the use of a password to which only the principal researcher and research assistants will have access. Finally, all the material and data will be kept for 5 years and then destroyed.

Peer review of the protocol required by the ethics committee is presented in Multimedia Appendix 3.

## Results

The project began in January 2016 with the ethics application for the 3 phases of the project. Ethical approval was received in November 2016. Thus, participants could not be recruited for 11 months. For phase 1, recruitment began in December 2016 and ended in September 2017. By August 2017, 38 community workers and HSSP had completed the online survey. In addition, 15 caregivers have been interviewed. A paper is being written to present the results of phase 1.

Phase 2 began in the spring of 2017 and ended in June 2018. All the co-design sessions have been completed. A prototype has been developed and is being improved following feedback from participants in the recent co-design sessions. Moreover, 3 papers are being written to present the results of phase 2.

Phase 3 of the project will take place from September 2018 to December 2018.

## Discussion

### Reminder of the Purpose of the Study

Keeping older persons at home largely depends on the help provided by caregivers. However, they need support to identify the needs of the older person they are assisting, their own needs, and the formal services available to meet them [26]. The goal of this multicenter project is to develop an eHealth instrument, which will facilitate this process of looking for help. Nonetheless, this project, following the process chosen by the research team, entails certain challenges.

### Challenges Met Until Now

One of the first challenges encountered was obtaining ethical approval. On the one hand, the multicenter nature of the project required obtaining a letter of support from each of the 11 organizations of the HSS network selected by the project. The presentation of the project and the different intermediaries and particularities for each organization were such that 11 months were necessary to complete the ethical process.

Another challenge was that of the recruitment of caregivers, this is, of course, a challenge common to other projects involving those kinds of persons [8]. Although more than 30 FMGs were contacted to request the participation of caregivers, this did not result in the recruitment of any participants. In addition to the fact that we do not have the confirmation that the FMGs actually posted the study project, we hypothesize that the caregivers do not recognize themselves as a caregiver or legitimate to bear this identity [26] and, therefore, do not feel concerned by the project unless the approach is straightforward. To date, the optimal method of recruitment has been through contact with community workers and HSSP. Nevertheless, on the one hand, this reduces the potential number of caregivers, and on the other hand, it leads to a bias in the selection process, due to the fact that these caregivers already have access to a formal service. Therefore, this is a limitation of the project, which needs to be taken into account during the analysis of the results.

### Anticipated Challenges

Moreover, the methodology selected to develop an eHealth tool, which genuinely responds to the needs of caregivers, is based on a co-design approach. To our knowledge, few studies have employed this methodology to develop an eHealth tool for caregivers. Co-design includes future users, in this case, caregivers, in the development of the tool, as co-designers. Therefore, this implies a sharing of power with people untrained in either research or design. This is a challenge not faced by other studies led only by research teams. The sharing of discourse and decision-making power is clearly a major challenge not only for researchers but also for future users [44]. As mentioned by Meiland [45,46], 1 of the risks of this method is that the users cannot express their own needs or ideas and instead may simply rally around the dominant figures in the group. To lessen this risk, 2 doctoral students will examine this aspect of co-design. The thesis of 1 of the doctoral students associated with this project bears on potentially unspoken elements in co-design sessions, and she will validate the data with individual interviews following the co-design sessions, if some elements of content were not raised. These potentially unspoken issues will be discussed in the working sessions with the advisory committee, to be able to take them into account, while obviously continuing to respect confidentiality. The second student’s thesis will bear, among other matters, on the hoped-for genuinely democratic process, which co-design approach can entail. Moreover, the role of the advisory committee is to ensure that the content emerging from the co-design sessions is effectively incorporated into the development of the tool and that it is not simply the preferences and expertise of researchers that prevail.

Another challenge of this project will be to ensure consistency among the different co-design sessions. Usually, the participants are the same from one session to another, which ensures fluidity between sessions and stimulates the acquisition of design skills. This will not be the case in this study. For this reason, previous group decisions should be presented at each new session to ensure participants’ understanding and consistency of decision making. In addition, the role of the advisory committee is to ensure consistency. Moreover, the choice of analyzing the data between each session is designed to also maintain this common thread.

Finally, 1 of the common challenges for all co-design projects is the design of a tool by those who do not necessarily possess any expertise in design or even basic computer skills. Among other things, 1 of the pitfalls encountered in this sense is the difficulty of envisaging new technologies or discussing abstract concepts, such as potential functionality, for example [35]. Among the solutions used to counter these problems is the use of prototypes (light and medium fidelity) to help participants visualize what is discussed [44].

eHealth can support caregivers of older persons in their process of seeking help. This study aims to develop this type of tool through a co-design methodology. Although there are some challenges associated with this type of methodology, it still remains relevant, because it really involves future users in the development of a tool that, in our opinion, increases the chances that it will meet their needs.

## Appendix

Multimedia Appendix 1 Online questionnaire for health and social services or community workers.

Multimedia Appendix 2 The interview plan of semidirected individual interviews with caregivers.

Multimedia Appendix 3 Scientific evaluation of the protocol.

## Abbreviations

AAHLS: All Aspects of Health Literacy Scale
eHealth: electronic health
FMG: family medicine group
HSS: health and social services
HSSP: health and social service professionals
IBM: International Business Machines Corporation
