# Supplementary material for: Supporting the Process of Help-Seeking by Caregivers of Functionally Dependent Older Persons Through Electronic Health: Protocol for a Multicenter Co-Design
Source: JMIR Res Protoc. 2019 Apr 26;8(4):e11634. doi: 10.2196/11634 (PMC6658263; doi:10.2196/11634)
Supplement: Multimedia Appendix 1 [file resprot_v8i4e11634_app1.pdf]

Multimedia Appendix

1. Online questionnaire for interveners (in French followed by an English translation)

French version

Il n'y a ni bonne ni mauvaise réponse. Vous devez simplement répondre spontanément selon ce que vous pensez.

MERCI DE VOTRE PARTICIPATION

Il y a 12 questions dans ce questionnaire.

Section 1 : Renseignements généraux. Les questions qui suivent portent sur votre situation professionnelle afin de mieux vous connaitre.

1. Indiquez votre genre

a) Féminin

b) Masculin

2. Indiquer le nom de votre poste actuellement occupé

3. Indiquer le nombre d’années que vous occupez ce poste

4. Indiquer votre région administrative

5. Indiquer votre organisation (lieu d’emploi)

Section 2 : Le processus de recherche d’aide. Les questions qui suivent portent sur les actions qui sont susceptibles de faciliter le processus de recherche d’aide du proche aidant.

6. a) Qu’est-ce qui, selon vous, permet à un proche de se reconnaitre comme un proche aidant?

6. b) Quelles actions pourraient aider le proche aidant à se reconnaitre comme tel?

7. Qu’est-ce qui, selon vous, aide le proche aidant à prendre conscience qu’il y a un problème?

8. Qu’est-ce qui, selon vous, facilite l’identification par le proche aidant des besoins de la personne qu’il soutient et de ses propres besoins?

9. Quels sont, selon vous, les éléments déclencheurs qui incitent les proches aidants à aller chercher de l’aide?

10. Qu’est-ce qui, selon vous, faciliterait la recherche de ressources par les proches aidants?

11. Qu’est-ce qui, selon vous, mobilise le proche aidant à prendre contact avec une ressource?

12. Est-ce qu’il y a un profil de proche aidant qui tarde davantage à chercher de l’aide? Si oui, lequel?

English version

There is no good or bad answer. You simply have to answer spontaneously according to what you think.

THANK YOU FOR YOUR PARTICIPATION

There are 13 questions in this questionnaire.

Section 1: General Information. The questions that follow relate to your professional situation in order to know you better.

1. Indicate your gender

a) Female

b) Male

2. Enter the name of your current position

3. Indicate the number of years you occupy this position

4. Indicate your administrative region

5. Indicate your organization (place of employment)

Section 2: The process of help- seeking. The following questions focus on actions that may facilitate the process of help seeking from caregivers.

6. a) What do you think enables a caregiver to recognize themselves as a caregiver?

6. b) What actions could help the caregiver recognize himself as such?

7. What do you think helps the caregiver to realize that there is a problem?

8. What do you think makes it easier for caregivers to identify the needs of the seniors they are caring and their own needs?

9. What do you think triggers family caregivers to seek help?

10. What do you think would make it easier for family caregivers to find resources?

11. What do you think mobilizes the caregiver to contact a resource?

12. Is there a family caregiver profile who wait too long in seeking help? If yes which?
