# Supplementary material for: Supporting the Process of Help-Seeking by Caregivers of Functionally Dependent Older Persons Through Electronic Health: Protocol for a Multicenter Co-Design
Source: JMIR Res Protoc. 2019 Apr 26;8(4):e11634. doi: 10.2196/11634 (PMC6658263; doi:10.2196/11634)
Supplement: Multimedia Appendix 2 [file resprot_v8i4e11634_app2.pdf]

Multimedia Appendix 2: Interview guide for individual interviews with caregivers - Phase 1

Introduction: project presentation and consent

<u>Section 1: Sociodemographic data (including the use of the Internet, tablets and smartphones). This section includes 16 questions.</u>

<u>Section 1 (a) General Information. The following questions are about your personal situation in order to know you better.</u>

1. What is your age?

2. Indicate your administrative region

3. What is your country of origin?

4. What is your level of education? For example, Secondary 1, College, University…

5. What is your annual family income approximately? (a) less than $20,000; (b) $ 20,000–34,999; (c) $ 35,000–49,999; (d) $ 50,000–74,999; e) more than $75,000

6. What is your relationship with your loved one (spouse, child, friend…)?

7. What health problems has your loved one encountered in recent years (memory problem, Parkinson's, stroke, …)?

8. How long have you been helping your loved one?

9. What task(s) do you do for your loved one?

10. How would you describe your state of health?

<u>Section 1 (b) Use of the Internet, tablets and smartphones</u>

1. Have you used the Internet in the last 12 months?

Yes indeed

b) No, Do you have access to a resource person if you need to use the internet? Who is it? Does she live with you?

If no, stay at b) Go to question 7

2. How many years have you been using the Internet?

3. In a typical month, how often do you use the Internet?

4. During a typical week, how many hours on average do you spend on the Internet?

5. In the last 12 months, with which device(s) did you have access to the Internet? (Computer, tablet, smartphone) Where did you use the Internet? Which one do you use most often?

6. For what purpose do you use the Internet? For example, to check your email, instant messaging, websites, social networks, search for information, read news, online shopping, games, music, film… See question 8.

7. Why do not you use the Internet? Dig if necessary…

8. What difficulties, if any, do you encounter with the technology (s) used?

Section 2: Process of seeking help from the caregiver. This section includes between 9 and 13 questions.

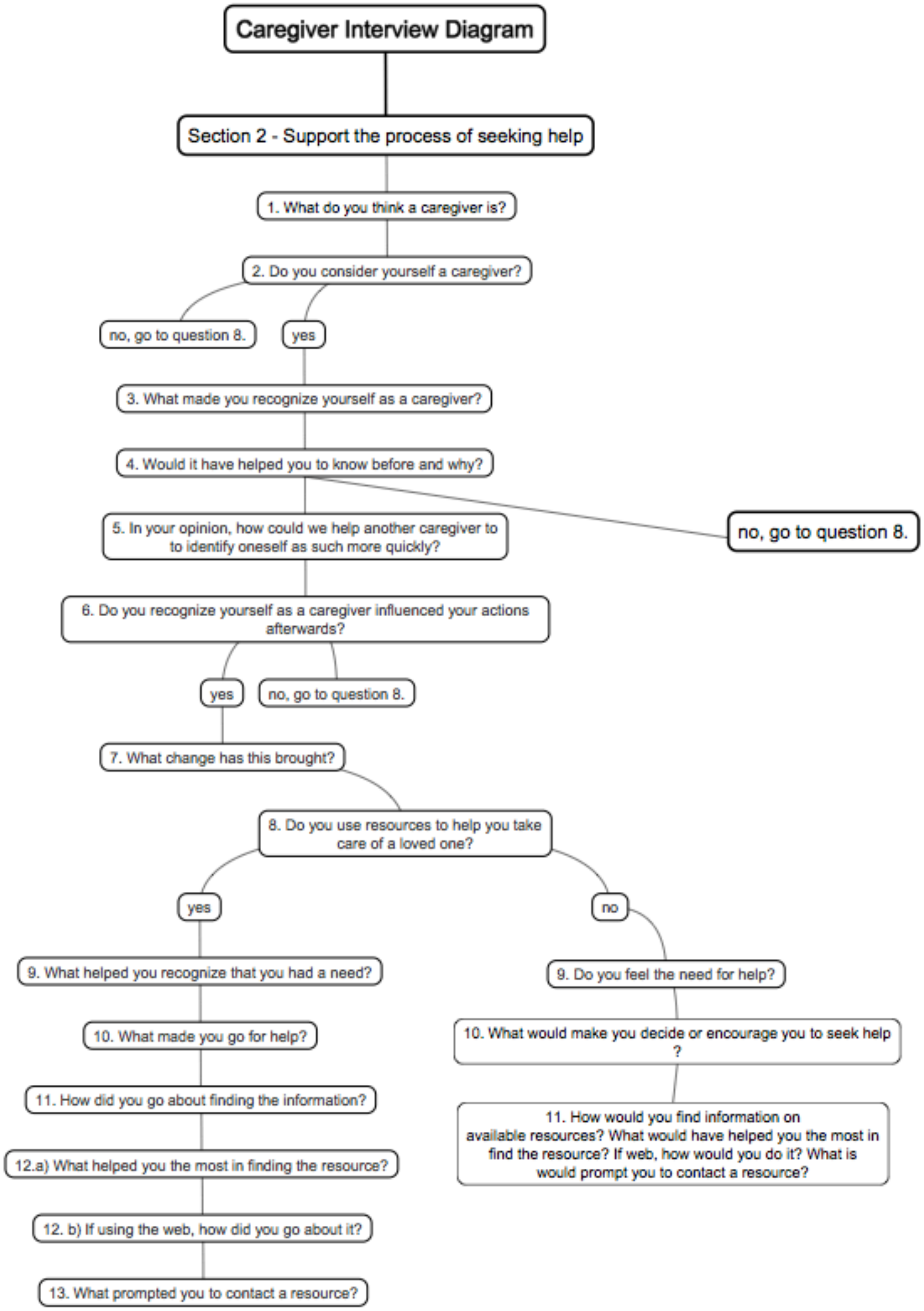

Section 3: All aspects of health literacy scale (AAHLS) (Chinn & McCarthy, 2013)
