# Supplementary material for: Supporting the Process of Help-Seeking by Caregivers of Functionally Dependent Older Persons Through Electronic Health: Protocol for a Multicenter Co-Design
Source: JMIR Res Protoc. 2019 Apr 26;8(4):e11634. doi: 10.2196/11634 (PMC6658263; doi:10.2196/11634)
Supplement: Multimedia Appendix 3 [file resprot_v8i4e11634_app3.pdf]

Centre de recherche
de l'Hôtel-Dieu de Lévis

# Rapport d'évaluation scientifique

| | |
|---|---|
| **Nom du chercheur principal** | Dominique Giroux |
| **Titre du projet** | Mieux répondre aux besoins des proches aidants pour un maintien à domicile sécuritaire des ainés en perte d'autonomie |
| **Numéro du projet de recherche** | |

## Résumé de l'évaluation

| | | | | |
|---|---|---|---|---|
| 1. Revue de littérature | ad. = | inad. = | rev. = | n/a = |
| 2. Objectif(s) du projet | ad. = X | inad. = | rev. = | n/a = |
| 3. Hypothèse(s) de l'étude | ad. = | inad. = | rev. = | n/a = X |
| 4. Participants | | | | |
| a. Taille de l'échantillon | ad. =X | inad. = | rev. = | n/a = |
| b. Critères d'inclusion/exclusion | ad. X= | inad. = | rev. = | n/a = |
| c. Modalités de recrutement | ad. = | inad. = | rev. =X | n/a = |
| 5. Instruments de mesure | ad. =X | inad. = | rev. | n/a = |
| 6. Collecte de données / Procédure(s) | ad. = | inad. = | rev. =X | n/a = |
| 7. Contrôle des variables confondantes | ad. = | inad. = | rev. = | n/a = X |
| 8. Méthodes d'analyse des résultats | ad. =X | inad. = | rev. = | n/a = |
| 9. Échéancier | ad. = X | inad. = | rev. = | n/a = |
| 10. Pertinence des retombées du projet | ad. = | inad. = | rev. = | n/a = |
| 11. Milieu de recherche | | | | |
| a. Chercheur principal | ad. = X | inad. = | rev. = | n/a = |
| b. Collaborateurs au projet | ad. = X | inad. = | rev. = | n/a = |
| c. Ressources prévues | ad. = X | inad. = | rev. = | n/a = |
| 12. Évaluation globale | | | | |
| a. Clarté et cohérence du texte | ad. =X | inad. = | rev. = | n/a = |
| b. Renseignements fournis | ad. = X | inad. = | rev. = | n/a = |

**Légende :** ad = adéquat(e) ; inad.= inadéquat(e) ; rev. = à revoir ; n/a = ne s'applique pas ; s/o = sans objet

## Commentaires

| | | | Commentaires |
|---|---|---|---|
| 1. | Revue de littérature | ☐ s/o | La recension des écrits est bien écrite et claire. Dans un futur outil pour guider les aidants, Est-ce qu'il serait pertinent d'inclure dans une approche préventive, d'encourager le dialogue entre l'aidé et l'aidant pour identifier tôt ce que les deux parties souhaitent faire ou ne pas faire comme tâches ou responsabilités à assumer selon les diverses étapes de la trajectoire de la maladie. Il me semble que cela aiderait l'aidant dans son analyse de besoin.<br>À ce sujet, le commentaire précédent pourrait paraitre peu pertinent si l'emphase est mise sur le besoin des aidants pour un outil soutenant leur **processus de recherche d'aide** et que l'on reconnaît les besoins au plan fonctionnel, santé, social des personnes âgées en perte d'autonomie et proche aidants, mais que ce n'est pas l'objet central du projet (si oui, je questionnerais l'absence de la personne aînée dans le processus). Comme c'est très connexe, on oscille entre les deux et parfois on perd le focus du projet. Comme le terme outil peut référer à différents objets, est-ce possible de donner un exemple de ce que pourrait être potentiellement votre outil? Je sais qu'il sera co-contruit avec vos participants, mais une illustration serait utile |
| 2. | Objectif(s) du projet | ☐ s/o | Très bien |
| 3. | Hypothèse(s) du projet | ☒ s/o | |
| 4. | Participants | | |
| | a. Taille de l'échantillon | ☐ s/o | ok |
| | b. Critères d'inclusion / exclusion | ☐ s/o | ok |
| | c. Recrutement | ☐ s/o | Je trouve judicieux que l'équipe de recherche se soucie d'avoir une variabilité de caractéristiques (proches aidants et intervenants), que ce soit à l'étape de consultation et d'élaboration de l'outil. Est-ce que l'on peut spécifier comment on va s'assurer de la variabilité, notamment on niveau de la littératie?<br>Bonne idée de mettre sur le dépliant la question permettant d'aider les personnes à s'identifier comme proche aidant<br>Si aucun ou peu d'intervenant se manifeste (ce que je ne souhaite pas), est-ce qu'une mesure de relance ou autre stratégie est prévue? |

| | | |
|---|---|---|
| 5. Instruments de mesure | ☐ s/o | |
| 6. Collecte de données / Procédure(s) | ☐ s/o | Il serait pertinent de clarifier l'étape 2 : 2Est-ce qu'on doit comprendre que chaque séance de co-design dans une région donnée va participer aux 8 séances expliquées à la page 11? Ou si chaque séance de co-design d'une région donnée correspond à l'une des 8 séances décrites à la page 11? Si c'est le deuxième scénario, comment on s'assure de la continuité? Si c'est le scénario 1, comment on s'assure de la rétention des participants<br>Définir le terme mock-up |
| 7. Contrôle des variables confondantes | ☒ s/o | |
| 8. Méthodes d'analyse des résultats | ☐ s/o | Ce qui est proposé est cohérent avec le devis et le type de données |
| 9. Échéancier | ☐ s/o | |
| 10. Pertinence des retombées | ☐ s/o | Les retombées sont exposées de façon générale, pas toujours propre au futur outil, c'est-à-dire, qu'une formation ou un programme d'intervention pourrait donner les mêmes retombées. Le lien avec l'autodétermination de l'aîné me semble peu évident compte tenu que ce n'est pas discuté dans la problématique et la recension |
| 11. Milieu de recherche | ☐ s/o | Très bien, équipe compétente |
| 12. Évaluation globale | ☐ s/o | Excellent projet |

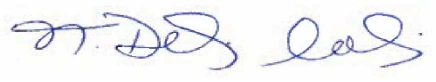

2016/08/15

Évaluateur (signature) Date
